# The cGAS/STING pathway in cancer: translating innate DNA sensing into therapeutic potential

**DOI:** 10.1172/JCI204552

**Published:** 2026-08-03

**Authors:** Yi Wang, Juan Angulo-Lozano, Yueqi Wang, Liang Deng

**Affiliations:** 1 Laboratory of Virology and Infectious Disease, The Rockefeller University, New York, New York, USA.; 2 Department of Urology, Weill Cornell Medical College, New York, New York, USA.; 3 Dermatology Service, Department of Medicine, and; 4 Human Oncology and Pathogenesis Program, Memorial Sloan Kettering Cancer Center, New York, New York, USA.; 5 Department of Dermatology, Weill Cornell Medical College, New York, New York, USA.

## Abstract

The cGAS/STING pathway is a central innate immune DNA-sensing system that links aberrant DNA species to innate immune and stress-response transcriptional programs and has emerged as a key regulator of tumor-immune interactions. In cancer, pathway outputs are shaped by interconnected downstream signaling modules, including type I IFN, NF-κB, autophagy, and stress-metabolic checkpoints, as well as by stringent spatial and biochemical regulation of both cGAS and STING. When activation is acute and appropriately compartmentalized, cGAS/STING signaling promotes antitumor immunity across multiple cellular compartments in the tumor microenvironment, supporting DC cross-priming and cytotoxic lymphocyte responses. In contrast, chronic or dysregulated activation rewires downstream signaling toward stress-adaptive and inflammatory programs that promote tumor progression, metastasis, and immune dysfunction, including deleterious effects in lymphocytes and the induction of suppressive myeloid and B cell populations. Here, we examine how context determines the consequences of cGAS/STING activation in cancer, review emerging therapeutic strategies that modulate this pathway, and discuss how its antitumor potential can be maximized while minimizing systemic toxicity and immune dysregulation.

## Introduction

The cyclic GMP-AMP synthase (cGAS)/stimulator of IFN genes (STING) pathway is a central innate immune DNA-sensing mechanism that enables cells to detect aberrant cytosolic DNA and mount inflammatory transcriptional programs. Early studies identified STING as an ER-resident adaptor required for type I IFN (IFN-I) induction downstream of cytosolic DNA and microbial stimuli (1). Subsequent work demonstrated that bacterial cyclic dinucleotides (CDNs) directly activate STING, establishing cyclic nucleotides as bona fide second messengers in innate immune signaling (2). A major advance followed with the identification of cGAS as the cytosolic DNA sensor that catalyzes the synthesis of the endogenous CDN 2′3′-cGAMP upon binding double-stranded DNA (3, 4). Together, these discoveries defined a conserved signaling axis linking DNA detection to IFN and inflammatory responses in antiviral and antibacterial immunity.

Although initially characterized primarily in host defense, cGAS/STING signaling is also intimately linked to tumor immunity. Tumor, stromal, and immune cells within the tumor microenvironment (TME) encounter diverse sources of mislocalized DNA and cGAMP, enabling pathway activation across multiple cellular compartments. In some contexts, cGAS-STING activation promotes antitumor immunity by inducing IFN-I, licensing dendritic cell (DC) cross-priming, and supporting cytotoxic T cell responses (5–7). However, accumulating evidence indicates that the same pathway can also promote tumor growth, metastasis, and resistance to therapy when engaged chronically or in distinct cellular and signaling contexts (8–10). These observations reveal a central paradox in cancer biology: cGAS/STING signaling can either reinforce immune surveillance or be co-opted to drive tumor progression, depending on how, where, and for how long it is activated.

In parallel with this mechanistic insight, the cGAS/STING pathway has emerged as an attractive therapeutic target in cancer. Multiple strategies to engage this axis have entered preclinical and clinical evaluation with the goal of enhancing antitumor immunity. Despite compelling preclinical rationale, early clinical trials of direct STING agonists have yielded only limited durable responses, underscoring our incomplete understanding of how pathway activation translates into therapeutic benefit in humans.

In this Review, we synthesize current understanding of how cGAS/STING signaling is regulated in cancer and how its divergent roles emerge from distinct biological regimes defined by activation duration, subcellular localization, and downstream transcriptional bias. Finally, we discuss emerging therapeutic strategies that seek to activate or tune cGAS/STING signaling in patients, emphasizing the need to balance immune activation with avoidance of maladaptive, tumor-promoting consequences.

## Core cGAS/STING signaling in cancer

In cancer biology, cGAS-STING is best viewed as a tunable signaling hub rather than a binary DNA sensor. Binding of cytosolic DNA enables cGAS to generate 2’3’-cGAMP, which activates STING and promotes its ER-to-Golgi trafficking, TBK1/IRF3 signaling, NF-κB activation, and downstream IFN and inflammatory programs (1, 3, 4, 11–20). The biological outcome depends on three coupled axes. The temporal axis distinguishes transient activation, which favors immune priming, from chronic activation, which favors adaptation and inflammation. The spatial axis distinguishes cytosolic DNA sensing, ER-Golgi STING signaling, nuclear cGAS functions, and extracellular cGAMP transfer. The output axis reflects the relative dominance of IFN-I, canonical or noncanonical NF-κB signaling, autophagy, ER stress, and metabolic remodeling (19–24).

Mislocalized endogenous DNA can arise from micronuclei, DNA damage, replication stress, defective nucleases, mitochondrial dysfunction, and therapy-induced genotoxic stress (8, 25–34). Mitochondrial permeability transition, oxidative damage, metabolic stress, or defective mitophagy can release mtDNA into the cytosol, where it activates cGAS/STING signaling (31–33, 35, 36). In tumors, mtDNA sensing can support immune priming by DCs, but sustained mitochondrial stress may also reinforce inflammatory and stress-adaptive programs within the TME (7, 37, 38). Thus, the source and persistence of DNA determine whether pathway activation is immunogenic or maladaptive.

Spatial control is imposed first at the level of cGAS. cGAS is frequently enriched in the nucleus, where chromatin tethering and nucleosome binding restrict enzymatic activation despite constant exposure to genomic DNA (39–47). Micronuclei generated by chromosomal instability can expose DNA after envelope rupture, but the chromatin state of micronuclear DNA, accessibility to cGAS, DNA length and composition, and competition with chromatin-associated factors determine whether a micronucleus becomes immunogenic (8, 25–28, 48). Indeed, productive sensing requires accessible cytosolic dsDNA, but compact, nucleosome-rich chromatin sequesters cGAS and recruits BAF, which competes with cGAS for DNA binding (44–47). Accordingly, micronuclei generated by ionizing radiation or replication stress can fail to activate cGAS-STING despite envelope rupture (28), reconciling observations that chromosomal instability–associated (CIN-associated) micronucleation can be either innately immunostimulatory or signaling silent (26, 27, 38).

Autophagy maintains cellular homeostasis through lysosomal recycling and can either suppress early tumorigenesis or support survival of established and metastatic tumors under metabolic stress (49). STING activation induces noncanonical autophagy by trafficking to the ER-Golgi intermediate compartment, where it promotes WIPI2- and ATG5-dependent LC3 lipidation, facilitating clearance of cytosolic DNA and pathogens (50). STING also enhances lysosomal biogenesis independently of TBK1 by activating TFEB, TFE3, and MITF, further promoting clearance of cytoplasmic DNA (51).

STING activity is similarly shaped by trafficking and termination. Productive signaling requires ligand-dependent STING movement from the ER to the Golgi, where palmitoylation, membrane composition, and TBK1 recruitment support signal propagation (14, 52–57). Termination depends on post-Golgi trafficking, endolysosomal degradation, ESCRT-dependent mechanisms, and autophagy-related processes that limit signal duration (58–60). Extracellular and intracellular cGAMP-degrading enzymes, including ENPP1 and poxin-related enzymes, further constrain paracrine STING activation (61–65). These spatial and biochemical checkpoints collectively set the threshold for regime switching in cancer.

## Antitumor functions of cGAS-STING in the TME

**Figure 1:**
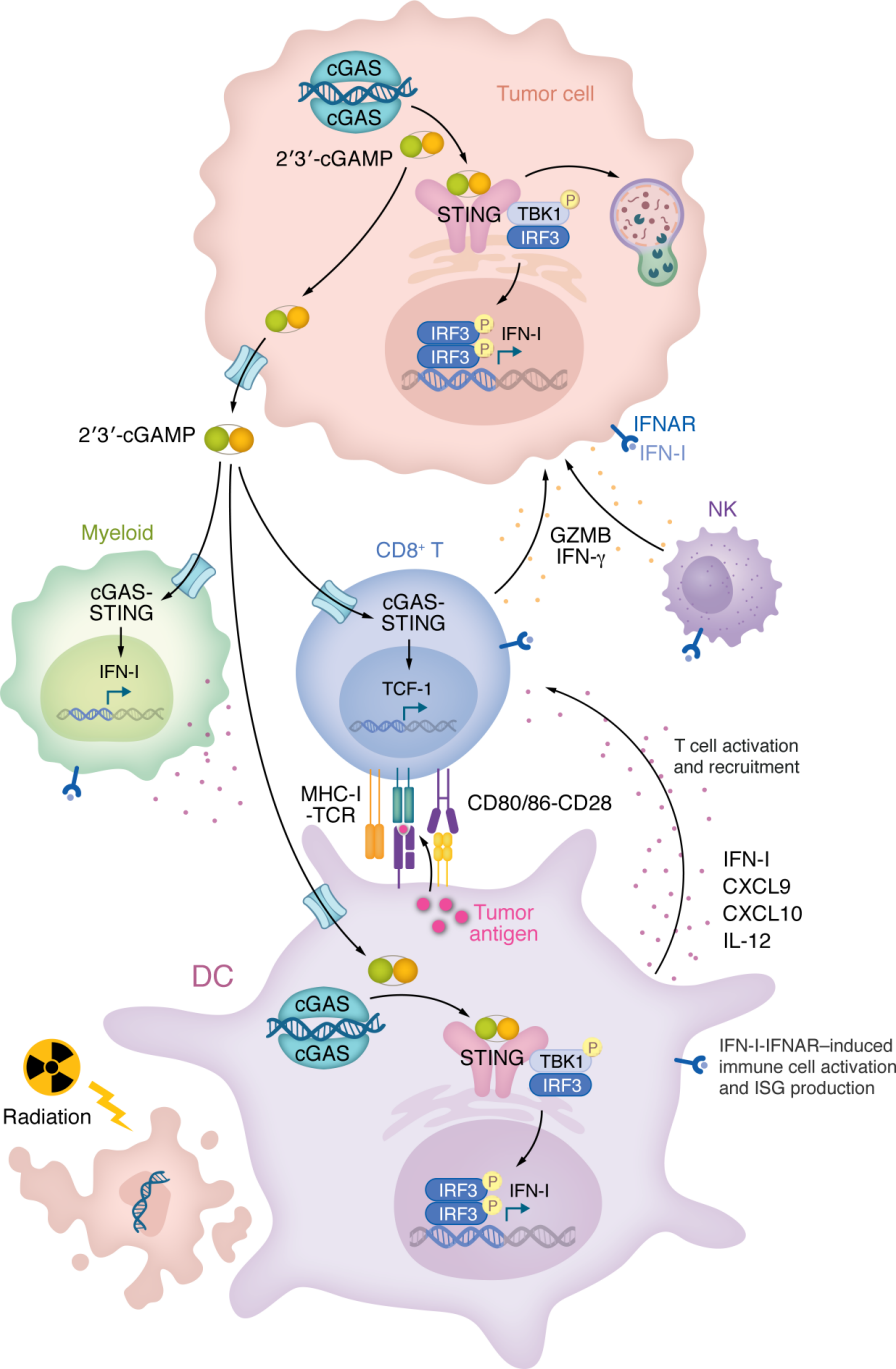
cGAS-STING–mediated orchestration of antitumor immunity. Aberrant DNA derived from genomic instability, replication stress, or genotoxic therapy (radiation, DDR inhibition) activates cGAS/STING signaling in tumor cells and cells of the TME. Acute, spatially restricted activation drives IFN-I, ISGs, and chemokine production (CXCL9, CXCL10) that recruit effector lymphocytes into the tumor. Tumor-derived cGAMP is exported through gap junctions, SLC19A1, the LRRC8/VRAC channel, and extracellular vesicles, enabling paracrine activation of STING in DCs, NK cells, and myeloid cells. In DCs, cGAS-STING activation licenses cross-presentation of tumor antigens on MHC-I and upregulates costimulatory molecules CD80/86, which together with MHC-I-TCR and CD28 engagement prime CD8^+^ T cells. NK and CD8^+^ T cells exert cytotoxic control through GZMB and IFN-γ, while T cell–intrinsic cGAS/STING signaling supports TCF-1^+^ stem-like CD8^+^ T cell maintenance. Coordinated, spatially restricted activation across these compartments converts genome instability into productive antitumor immunity.

The cGAS/STING pathway plays a central role in shaping antitumor immunity by linking aberrant DNA species to innate immune activation and adaptive immune priming. Within the TME, cGAS/STING signaling operates across tumor cells, antigen-presenting cells, and lymphocytes, where its spatial localization and activation context determine immunological outcomes (Figure 1).

### DCs as central amplifiers of cGAS-STING–mediated antitumor immunity.

IFN-I and host IFN-I signaling are indispensable for effective antitumor immunity (7). Among immune cells in the TME, DCs, especially type 1 conventional DCs (cDC1s), serve as the principal amplifiers of cGAS-STING–dependent antitumor responses by translating innate DNA sensing into adaptive T cell priming (5, 6, 66, 67). cDC1s are specialized for cross-presentation of tumor-derived antigens on MHC-I molecules and are essential for initiating and sustaining CD8^+^ T cell–mediated antitumor immunity (7, 68–70). Tumor DNA sensing by DCs via cGAS-STING occurs through multiple, partially redundant mechanisms, including phagocytosis of tumor-derived DNA and uptake of extracellular vesicles (5, 66, 71–73). In addition, intercellular transfer of cGAMP through gap junctions, transporter SLC19A1, or the LRRC8/VRAC channel provides a DNA-independent route for pathway activation within the TME (71–73).

STING signaling in DCs induces robust IFN-β production, upregulation of costimulatory molecules, and expression of chemokines such as CXCL9 and CXCL10, which recruit effector T cells into tumors (5, 6). In addition, STING activation in cDC1s functionally licenses production of IL-12, a key cytokine required for optimal CD8^+^ T effector cell differentiation and durability (74). STING activation also induces NF-κB signaling by recruiting TBK1 in an IFN-I–independent manner, which promotes DC activation and confers antitumor immunity (17). Genetic ablation of cGAS or downstream signaling components in host DCs markedly impairs antitumor immunity and abrogates responses to immune checkpoint blockade (75).

Recent work has further revealed that immune checkpoint receptors expressed on DCs actively regulate cGAS-STING activation thresholds. Inhibitory receptors such as TIM-3 suppress uptake of extracellular DNA by intratumoral DCs, thereby limiting cGAS activation and IFN-I production (76). Antibody-mediated blockade of TIM-3 restores DNA uptake, unleashes DC-intrinsic cGAS/STING signaling, enhances IFN-I output, and promotes CD8^+^ T cell infiltration and tumor control, identifying DCs as both sensors and regulatory gatekeepers of cGAS-dependent antitumor immunity (76).

### Tumor-intrinsic cGAS-STING: DNA damage, chromosomal instability, and immunogenicity.

Tumor cells exhibit oncogene activation, replication stress, defective DNA repair, and mitotic errors, generating cytosolic DNA species that can engage cGAS. Acute tumor-intrinsic cGAS activation leads to production of cGAMP, induction of IFN-I, and upregulation of IFN-stimulated genes (ISGs), contributing to tumor immunogenicity. Mismatch repair-deficient (dMMR) tumors exemplify this mechanism (77, 78). High mutation burden and persistent DNA damage in dMMR cells lead to enhanced cytosolic DNA accumulation, cGAS activation, and IFN-I production, which in turn promote antigen presentation, chemokine expression, and CD8^+^ T cell recruitment (77, 78). Loss of tumor-intrinsic cGAS in this setting reduces immune infiltration and confers resistance to immune checkpoint blockade (77).

In addition to innate immune sensing, cGAS/STING signaling is essential for the induction of senescence-associated secretory phenotype (SASP) genes in response to DNA damage, linking DNA sensing to the growth arrest that can contribute to tumor suppression (79). cGAS-STING–engaged autophagy, triggered by telomeric damage, acts as a final barrier to prevent oncogenic transformation of fibroblasts or epithelial cells that escape senescence during replicative crisis (80). Therefore, inhibition of cGAS/STING signaling or autophagy enables cells in crisis to bypass cell death and continue proliferating despite accumulating CIN (80).

Beyond primary tumor immunogenicity, tumor-intrinsic STING signaling can also function as a metastasis-suppressive checkpoint. In a model of lung adenocarcinoma metastasis, STING signaling acts as a suppressor of metastatic outbreak during exit from dormancy, with STING activity increasing in metastatic progenitors as they reenter the cell cycle (81). Breakthrough metastases showed attenuated STING activity via hypermethylation of STING regulatory elements, whereas dormancy reentry driven by TGF-β was associated with repressed expression of STING (81). Systemic STING agonist treatment eliminated dormant metastases and prevented spontaneous outbreaks in a T cell– and NK cell–dependent manner, and these effects required tumor-intrinsic STING (81).

Importantly, tumor-intrinsic cGAS signaling does not primarily act through direct tumor cell killing. Instead, its dominant contribution lies in converting tumor cells into sources of innate immune ligands that license immune activation. This function is particularly relevant following genotoxic therapies, which increase cytosolic DNA burden and transiently amplify cGAS/STING signaling. When appropriately timed and spatially constrained, such activation may enhance antigen cross-presentation and synergize with T cell–based immunotherapies.

The asymmetry between cGAS and STING in tumor versus host compartments reflects the directional flow of cGAMP. Tumor cGAS is required to generate cGAMP from cytosolic DNA, but tumor STING is largely dispensable because cGAMP is exported and sensed by host STING in DCs (82–86). Conversely, host STING is essential as the dominant adaptor in DCs that translates tumor-derived cGAMP into IFN-I and CD8^+^ T cell priming (5, 7), whereas host cGAS is partially redundant because immune cells can acquire tumor-derived cGAMP rather than synthesize it themselves (82). This asymmetry explains why tumor cGAS plus host STING define the minimal genetic requirement for productive antitumor responses (82, 83).

### T cell–intrinsic cGAS-STING in supporting T cell fitness.

Beyond antigen-presenting cells, cGAS/STING signaling within T cells contributes to antitumor immunity by regulating T cell fitness, persistence, and differentiation state. cGAS/STING signaling in CD8^+^ T cells supports maintenance of a TCF-1^+^ stem-like population that sustains long-term effector responses (87). Mechanistically, cGAS/STING signaling in T cells induces autocrine IFN-I signaling that restrains Akt activation, preserving metabolic flexibility and preventing T cell exhaustion. Genetic deletion of cGAS or STING in T cells compromises their proliferative capacity, persistence after adoptive transfer, and overall antitumor efficacy in vivo (87). Another recent study showed that cGAMP produced from irradiated cancer cells taken up by CD8^+^ T cells is crucial for their antitumor functions, although the difference between autocrine versus paracrine cGAS/STING signaling was not clear (88). These findings position cGAS not only as a sensor of danger but also as a rheostat tuning T cell intrinsic programs that determine durability of antitumor immunity.

Notably, T cell–intrinsic cGAS/STING signaling is bifunctional. Low-amplitude, transient signaling driven by endogenous DNA or paracrine cGAMP supports TCF-1^+^ stem-like CD8^+^ T cells and metabolic fitness via autocrine IFN-I (87, 88), whereas high-amplitude or sustained STING activation disrupts ER calcium homeostasis and triggers mitochondrial dysfunction and cell death (21, 85, 89, 90). T cell state also matters. Chronically stimulated or exhausted T cells are more susceptible to STING-mediated cell death than naïve cells, and tumor-derived DNA or extracellular vesicles can bias T cell–intrinsic signaling toward FoxP3 induction (85). T cell–intrinsic cGAS-STING is thus a tunable input rather than a uniformly beneficial or detrimental program.

### NK cells, macrophages, endothelium, and tertiary lymphoid structures.

cGAS/STING signaling also influences antitumor immunity across additional stromal and immune compartments. In NK cells, cGAS activation by tumor-derived cGAMP enhances cytotoxic function and cytokine production, in part by promoting IFN-I–dependent transcriptional programs (82).

In nonhematopoietic stromal cells, including endothelial cells, cGAS/STING signaling contributes to vascular activation and immune cell recruitment (91). Activation of endothelial cGAS-STING promotes expression of adhesion molecules and chemokines, facilitating lymphocyte trafficking into tumors (91). Endothelial cGAS-STING activation also reshapes tumor vasculature. STING agonists normalize tumor vessels and promote pericyte coverage and adhesion molecule upregulation in an IFN-I– and CD8^+^ T cell–dependent manner, an effect requiring endothelial STING and synergizing with VEGFR2 blockade and immune checkpoint inhibitors (92, 93). Conversely, sustained tumor cell–intrinsic cGAS/STING signaling may support pathological angiogenesis through chronic NF-κB–driven cytokine programs (8, 91). Furthermore, recent work suggests that cGAS/STING signaling may support the formation and maintenance of tertiary lymphoid structures, which correlate with improved responses to immunotherapy in multiple cancer types (94).

Together, these findings establish cGAS-STING as a distributed innate immune pathway whose antitumor efficacy emerges from coordinated activation across tumor, immune, and stromal compartments.

## Protumor, prometastatic, and immunosuppressive roles

**Figure 2:**
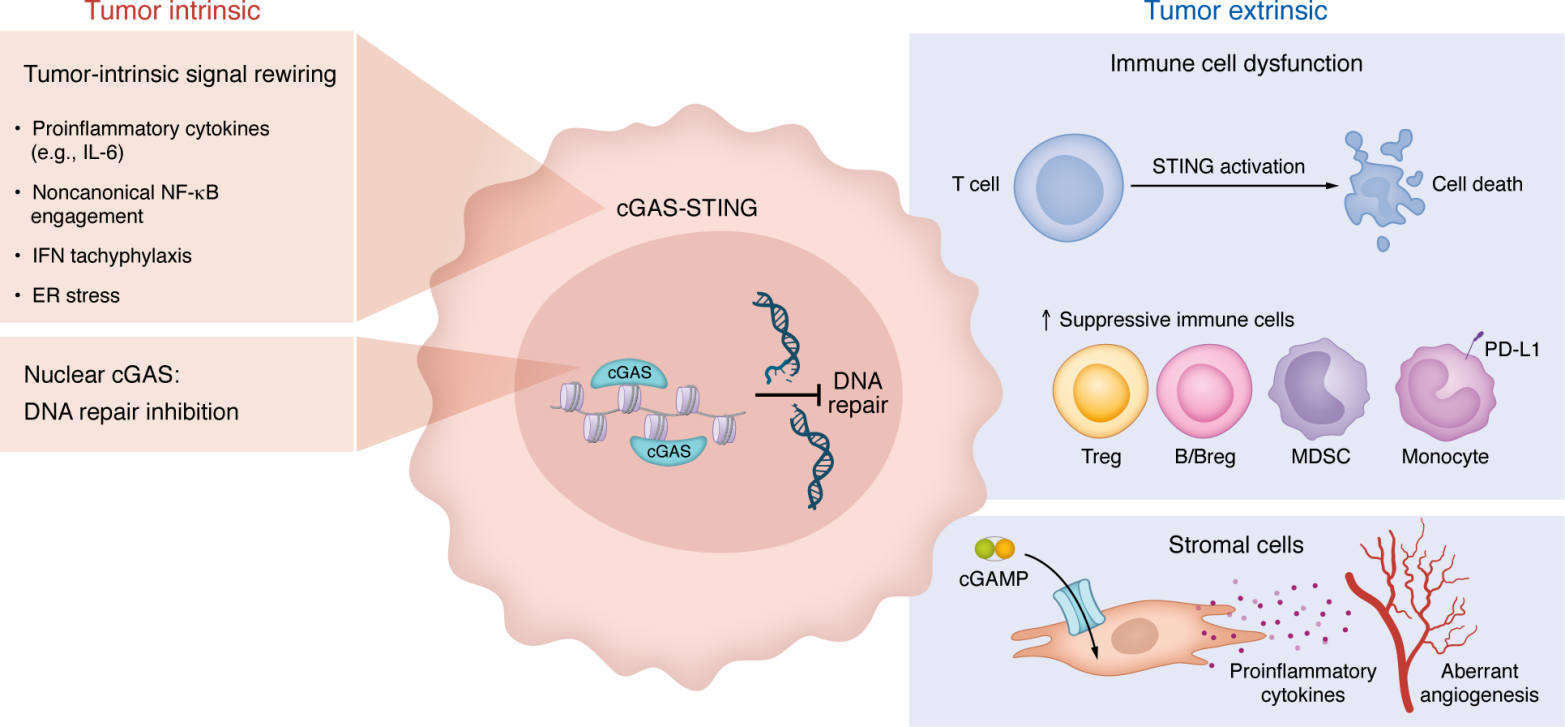
Protumorigenic cGAS/STING signaling in cancer. Persistent cGAS-STING signaling driven by ongoing chromosomal instability and chronic micronucleation shifts pathway output away from beneficial IFN-I responses toward noncanonical NF-κB, ER stress, and proinflammatory programs. Tumor cell–intrinsic effects include the following: chronic STING signaling that promotes IL-6 and other proinflammatory cytokines, noncanonical NF-κB engagement, IFN tachyphylaxis, ER stress and autophagy, and epithelial-mesenchymal transition/invasion/metastasis programs. In addition, nuclear cGAS engages chromatin and inhibits homologous recombination by suppressing PARP1 and RAD51, exacerbating genomic instability independently of STING. Tumor-extrinsic effects include changes in immune cells. Within T cells, cytoplasmic STING activation triggers calcium dysregulation and apoptosis and cGAS/STING signaling skews the surrounding immune compartment toward suppressive populations, including FoxP3^+^ Tregs, IL-10/IL-35–producing regulatory B cells (Bregs), MDSCs, and PD-L1^+^ monocytes. Within stromal and vascular niches, cGAMP uptake by cancer-associated fibroblasts drives chronic IL-6 and CCL2 production, and persistent endothelial signaling supports aberrant angiogenesis and an immunosuppressive vascular niche. Together, these mechanisms convert innate DNA sensing into a driver of tumor progression and immune dysfunction.

A paradox here is that the genes encoding cGAS and STING are rarely lost in cancers (95). Although acute, spatially restricted activation of the cGAS/STING pathway can initiate antitumor immunity, chronic or dysregulated signaling promotes tumor progression, metastasis, and immune dysfunction. These divergent outcomes arise from distinct signaling regimes shaped by CIN, activation duration, downstream transcriptional bias, and cellular compartmentalization (Figure 2).

### CIN-driven rewiring of cGAS/STING signaling.

CIN is a defining feature of advanced malignancies and results in persistent formation of micronuclei and cytosolic DNA species (8, 25, 95). While micronuclear rupture can initiate innate immune alerting, CIN also leads to repeated cGAS engagement and proinflammatory responses in cancer cells (8, 10, 96, 97).

STING activation was first implicated in promoting tumorigenesis in a skin cancer model, in which carcinogen treatment activated STING signaling in both hematopoietic and nonhematopoietic cells and led to proinflammatory cytokine production (9). Later studies delineated that in metastatic tumors, prolonged activation driven by CIN preferentially engages noncanonical NF-κB rather than IFN-I or canonical NF-κB signaling (8). Increased nuclear translocation of RelB drives transcriptional programs associated with epithelial-mesenchymal transition (EMT), invasion, and metastatic dissemination (8). In triple-negative breast cancer, cGAS-STING activation induces IL-6 production and subsequently initiates STAT3 signaling to protect cancer cells from CIN-induced cell death, whereas IFN-I primarily activates a STAT1-dependent cell death pathway (10).

A central question is how CIN-driven cGAS/STING signaling transitions from IFN-I–mediated antitumor immunity to tumor-promoting inflammation. While cGAS/STING signaling activates senescence to restrict tumorigenesis, it also elicits a chronic proinflammatory response in cells that escape senescence and impairs immune surveillance (96). Chronic STING activation also results in IFN tachyphylaxis, a phenomenon characterized by decreased responsiveness to IFN-I following repeated stimulation (97). In vitro, repeated cGAMP treatment blunts IFN-I responses in cancer cells but later activates ER stress pathways (97). The upregulated ER stress response is also observed in CIN-high tumors in vivo and is associated with increased infiltration of suppressive immune cells (97). In this context, cGAS-STING functions as an adaptive pathway that enables tumor cells to exploit inflammatory signaling to support invasion and dissemination.

### Nuclear cGAS impairs DNA repair, promoting tumor evolution.

In addition to cytosolic DNA sensing, cGAS also promotes genome instability and tumorigenesis independently of STING signaling. In response to genotoxic stress, cGAS translocates into the nucleus and is recruited to sites of DNA damage, where it impedes formation of the PARP1-TIMELESS complex, thereby suppressing homologous recombination–mediated (HR-mediated) repair (98). This activity does not require downstream IFN signaling and instead reflects a physical competition between cGAS and core DNA repair machinery. By constraining HR, nuclear cGAS biases repair toward error-prone outcomes that exacerbate CIN. Notably, this DNA-repair inhibitory function is not evolutionarily conserved across all mammals: the naked mole rat cGAS ortholog lacks HR-suppressive activity due to sequence alterations that weaken chromatin engagement and instead promotes recruitment of DNA repair factors, thereby enhancing repair efficiency (99). This contrast underscores that suppression of DNA repair by cGAS is a tunable property rather than an obligate function and highlights that selectively modulating nuclear cGAS functions, rather than global pathway activation, could influence tumor evolution independent of immune signaling. A direct corollary is that cGAS depletion or pharmacologic inhibition should sensitize tumors to PARP inhibitors by relieving cGAS-mediated suppression of HR (98). Preclinical data support this concept (100–103), but clinical validation of this approach remains incomplete.

### cGAS-STING activation-induced immune dysfunction.

The dual roles of cGAS-STING in lymphocytes, particularly T cells, remain largely understudied. T cells exhibit poor tolerance to STING activation and readily undergo cell death (89, 90). STING activation disrupts calcium homeostasis and mitochondrial integrity, impairing proliferation and survival independently of canonical IFN signaling, thereby limiting cytotoxic T cell responses within tumors (21). Tumor-derived DNA or extracellular vesicles can activate T cell–intrinsic STING signaling to promote FoxP3 induction and Treg differentiation, thereby reducing CD8^+^ T cell infiltration and antitumor activity (84). In addition, systemic or tumor-associated STING activation expands IL-35–producing regulatory B cells, which suppress NK cell proliferation and cytotoxic function, facilitating immune evasion and tumor progression (85).

Radiation-induced activation of STING drives recruitment of monocytic myeloid-derived suppressor cells (MDSCs) into tumors. These cells suppress antitumor immunity and contribute to resistance to radiation and tumor progression, an effect that is abrogated by blocking STING-dependent chemokine pathways such as CCR2-mediated MDSC infiltration in mouse models (86). In tumors with low antigenicity, STING-dependent DNA sensing can enhance immunosuppressive states in the microenvironment, for example through induction of metabolic and cytokine programs that increase tolerance rather than elimination, supporting tumor growth (104). Moreover, STING activation in tumor monocytes has been linked to the upregulation of PD-L1, which promotes resistance to STING agonist therapy by creating a more suppressive and tolerogenic niche for tumor cells (105).

Beyond immune cells, cGAS/STING signaling can be engaged in stromal cells to support tumor progression. In brain metastasis, carcinoma cells form gap junctions with astrocytes that enable direct transfer of cGAMP into astrocytes, leading to STING activation and production of inflammatory cytokines, including IFN-α and TNF (106). These astrocyte-derived signals act back on metastatic tumor cells to activate STAT1 and NF-κB pathways, enhancing tumor cell survival and chemoresistance within the brain microenvironment (106).

Collectively, these studies establish that cGAS-STING activation across multiple immune compartments can converge on immunosuppressive and tolerogenic programs that blunt antitumor immunity and actively promote cancer progression.

## Therapeutic targeting of cGAS-STING in cancer

**Figure 3:**
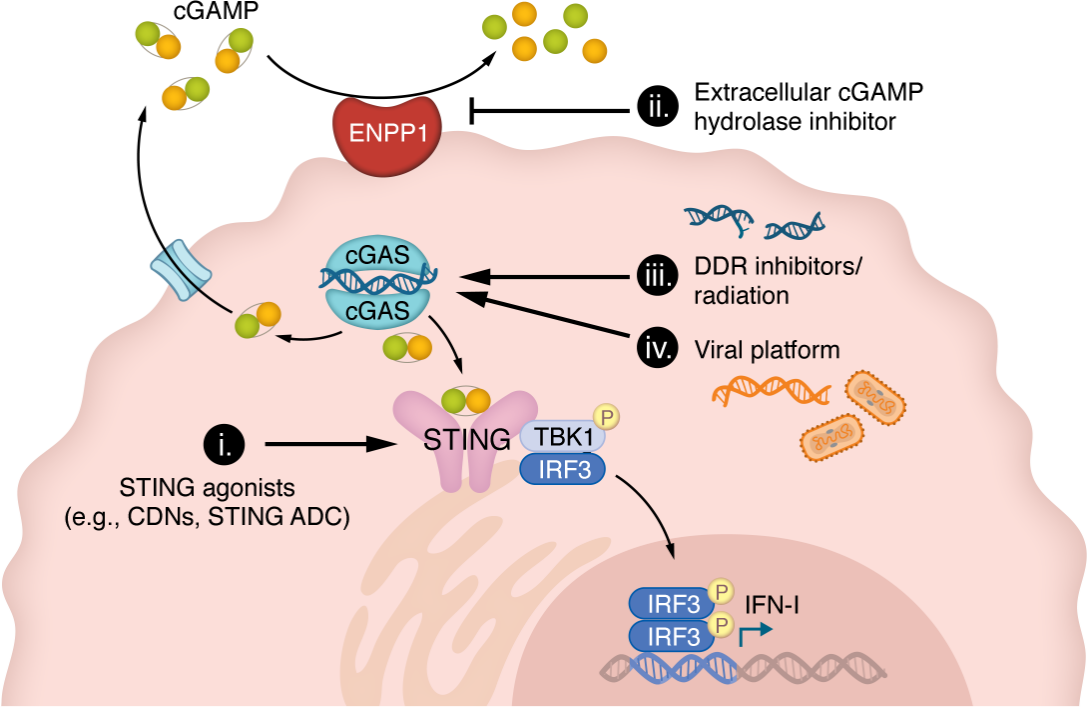
Therapeutic targeting of cGAS-STING for cancer treatment. Multiple strategies aim to harness cGAS/STING signaling for antitumor immunity, each engaging a distinct node of the pathway. (i) Direct STING agonists, including CDNs, non-CDN small molecules, and tumor-targeted ADCs engage STING directly to induce IRF3- and NF-κB–driven transcription. (ii) Extracellular cGAMP hydrolase inhibitors blocking ENPP1 prevent degradation of tumor-derived cGAMP in the extracellular space, sustaining paracrine STING activation. (iii) DDR inhibitors and radiation (PARPi, ATRi, CHK1i, ionizing radiation) generate cytosolic DNA and micronuclei that activate tumor-intrinsic cGAS, supplying endogenous cGAMP to license host STING. (iv) Viral platforms such as T-VEC and other oncolytic viruses provide foreign DNA that activates cGAS in infected and bystander cells. Convergent activation of the cGAS-STING-IRF3/NF-κB axis induces IFN-I and ISGs expression. The clinical utility of these strategies depends on tuning the magnitude, duration, and cellular compartmentalization of activation to achieve durable immune engagement while limiting chronic inflammation and species-specific differences in human STING responsiveness.

The cGAS/STING pathway has emerged as an attractive therapeutic target in oncology owing to its capacity to induce IFN-I, activate DCs, and promote cross-priming of cytotoxic T cells. However, early clinical experience revealed that indiscriminate pathway activation alone is often insufficient to generate durable systemic antitumor immunity. These findings have prompted diversification beyond direct agonists toward strategies that amplify endogenous cGAMP signaling, induce tumor-intrinsic DNA sensing through genomic stress, or engage broader innate immune networks through viral and oncolytic platforms (Figure 3 and Tables 1 and 2). These therapeutic strategies can therefore be broadly organized into four categories: direct STING agonists, amplification of extracellular cGAMP signaling, tumor-intrinsic activation through DNA damage, and viral/oncolytic platforms. The trials discussed below and summarized in Tables 1 and 2 were selected as representative examples to illustrate these mechanistic principles and are not intended as an exhaustive survey of cGAS-STING–related clinical studies.

### Direct STING agonists.

Early therapeutic efforts to directly activate STING employed synthetic CDNs, designed to mimic endogenous 2’3’-cGAMP following intratumoral administration. The prototypic CDN agonists ADU-S100 (MIW815) and ulevostinag (MK-1454) demonstrated clear pharmacodynamic evidence of STING pathway engagement, including induction of inflammatory cytokines, ISGs, and peripheral immune activation (107–110). However, objective responses remained limited in advanced solid tumors. In the first-in-human ADU-S100 study, paired tumor biopsies did not reveal substantial on-treatment changes in immune infiltration or RNA expression despite systemic immune activation (107). Combination of intratumoral CDNs with PD-1 blockade modestly improved response rates in selected tumor types, including triple-negative breast cancer, but durable systemic control and abscopal responses remained relatively infrequent (109).

To overcome the pharmacokinetic and delivery limitations of intratumoral CDNs, nonnucleotide small-molecule STING agonists have been developed with improved bioavailability and systemic dosing potential. SNX281 activates STING through ligand-induced dimerization within the STING binding pocket, and early clinical studies demonstrated on-target cytokine induction and manageable tolerability, although robust objective clinical responses have not yet been reported (111). E7766, a macrocycle-bridged pan-genotypic STING agonist designed for broader tumor accessibility, demonstrated pharmacodynamic cytokine induction and occasional disease stabilization in a first-in-human phase I/Ib trial in patients with relapsed or refractory solid tumors, but dose-limiting toxicities and a narrow therapeutic window were observed (112). While systemic delivery addresses the spatial constraints of intratumoral administration, achieving durable antitumor immunity without excessive inflammatory toxicity remains a major translational challenge.

A conceptual advance is the development of antibody-drug conjugates (ADCs) carrying STING agonist payloads to impose spatial control over innate immune activation. HER2-targeted XMT-2056 demonstrated tumor-localized STING activation with reduced systemic cytokine induction compared with free agonists and induced type III IFN programs and bystander immune activation, suggesting that antigen-targeted delivery may improve the therapeutic index of STING agonism (113, 114). DS3610, a STING ADC with novel Fc modifications, recently entered a first-in-human phase I trial in advanced solid tumors (NCT07159126).

Across this class, a consistent pattern has emerged: pharmacologic STING activation reliably induces measurable innate immune responses but rarely translates into durable tumor control. Several mechanisms likely contribute. Intrinsic pathway termination, through lysosomal trafficking, autophagy-dependent degradation, and progressive desensitization of IFN-I responses following repeated stimulation, limits the duration of productive immune priming. Counterregulatory programs, including PD-L1 upregulation (105), Treg infiltration (115), and recruitment of suppressive myeloid populations (86), further attenuate downstream immune responses. Species differences in STING biology also complicate translation: common human STING haplotypes such as HAQ and R232H respond suboptimally to bacterial CDNs that potently activate murine STING (116, 117), and human and mouse DC populations show divergent IFN output and viability following STING activation (118). Together, these observations underscore that the biological outcome of STING activation depends not simply on pathway engagement, but on the spatial, temporal, and cellular context in which signaling occurs.

### Amplifying paracrine STING: targeting extracellular cGAMP.

Tumor cells with CIN can generate and export cGAMP, which activates STING in neighboring DCs and macrophages, enabling tumor-derived cGAMP to trigger IFN-I production in bystander immune cells even when tumor cells themselves have silenced STING (119). ENPP1 degrades extracellular cGAMP and limits this intercellular signaling (62). Tumor-associated ENPP1 expression suppresses paracrine STING activation and promotes immune evasion in preclinical cancer models (120–122). Genetic or pharmacologic ENPP1 inhibition increases extracellular cGAMP levels, enhances ISG expression, and synergizes with radiation or immune checkpoint blockade in murine models (120–122). An orally bioavailable ENPP1 inhibitor (ISM5939), which enhanced extracellular cGAMP stabilization and antitumor activity in preclinical murine models (123), is being evaluated in a first-in-human phase I clinical trial (NCT06724042), although pharmacodynamic and efficacy data are not yet available. Conceptually, this strategy amplifies endogenous tumor-derived danger signals rather than directly activating STING. Whether such paracrine enhancement can produce durable clinical benefit remains to be determined.

### Tumor-intrinsic STING: radiation and DNA damage response inhibitors.

An indirect strategy to engage cGAS-STING is to exploit tumor-intrinsic DNA damage and genomic instability to generate cytosolic DNA ligands. Radiotherapy (RT) induces double-strand breaks and mitotic progression-associated micronuclei formation, leading to cytosolic DNA exposure and activation of the cGAS-STING-IFN-I axis (26, 27). Preclinical studies demonstrate that intact STING signaling is required for optimal RT-induced immune priming and synergy with immune checkpoint blockade (66). Importantly, this response is dose dependent: high radiation doses can induce the exonuclease TREX1, limiting cytosolic DNA accumulation and attenuating cGAS-STING activation (124).

Clinically, the randomized PEMBRO-RT trial showed that addition of stereotactic body RT (SBRT) to the PD-1 inhibitor pembrolizumab improved response rate and progression-free survival in metastatic non–small cell lung cancer (NSCLC) (125), consistent with RT functioning as an immunologic primer. However, STING pathway biomarkers were not assessed, so the contribution of cGAS/STING signaling to clinical benefit remains inferential. Broader clinical experience combining RT with immune checkpoint blockade highlights heterogeneous outcomes that depend on dose, fractionation, timing, and tumor immune contexture, underscoring the need for direct pharmacodynamic assessment of innate sensing pathways in future trials (126).

DNA damage response (DDR) inhibitors provide another route to innate priming by increasing unresolved DNA lesions and cytosolic DNA. PARP inhibition generates cytoplasmic chromatin fragments that engage cGAS-STING, induce IFN-I signaling and CCL5, and promote DC-mediated T cell recruitment (100–102). In BRCA1-deficient triple-negative breast cancer models, olaparib-induced T cell recruitment was mediated through tumor cell–intrinsic activation of cGAS-STING with subsequent paracrine activation of DCs, and CRISPR-mediated deletion of STING in tumor cells abrogated olaparib-induced T cell infiltration (102). These findings suggest that tumor cGAS-STING competence, HR deficiency status, baseline IFN signatures, and immune infiltration may help stratify patients for PARP inhibitor-immunotherapy combinations.

Clinically, combinations of PARP inhibitors with immune checkpoint blockade have demonstrated safety and preliminary efficacy. In the MEDIOLA trial, olaparib plus durvalumab achieved a 92.2% objective response rate (ORR) in germline BRCA-mutated platinum-sensitive relapsed ovarian cancer, and olaparib plus durvalumab and bevacizumab demonstrated encouraging activity in nongermline BRCA-mutated patients (127). Translational analyses from the breast cohort demonstrated upregulation of IFN-I, STING, and JAK/STAT pathway gene signatures in 5 of 6 patients on olaparib, consistent with tumor-intrinsic innate immune engagement (128). Similarly, the TOPACIO/KEYNOTE-162 study showed activity of niraparib plus pembrolizumab in triple-negative breast and recurrent ovarian cancer, with response rates of 18%–21% in unselected populations (129, 130).

Ataxia telangiectasia and Rad3-related (ATR) inhibitors intensify replication stress and CIN, increasing micronuclei formation and promoting cGAS-STING-IFN-I activation in a context-dependent manner (131, 132). Preclinical work in small cell lung cancer demonstrated that ATR inhibition directly activates tumor cell–intrinsic cGAS/STING signaling, leading to cytosolic DNA accumulation, IFN induction, and enhanced sensitivity to immune checkpoint blockade (133). Both DNA- and RNA-sensing pathways may contribute to ATR inhibitor–enhanced IFN signaling following RT, with pathway dominance varying by tumor type (134).

The heterogeneous clinical outcomes observed across direct STING agonists, DDR inhibitor combinations, and RT-based strategies reflect a common unresolved challenge: the absence of validated biomarkers to prospectively identify patients likely to benefit. Tumor-intrinsic STING pathway competence and baseline IFN signatures are most relevant to direct agonists, which require an intact, nondesensitized pathway to produce durable priming. CIN status and ENPP1 expression may identify tumors that generate and export sufficient endogenous cGAMP to benefit from paracrine amplification through ENPP1 inhibition. HR deficiency status and radiation dose relative to the TREX1 activation threshold are the corresponding determinants for DDR inhibitor combinations and RT, respectively, where the relevant variable is the capacity to generate and sustain cytosolic DNA. Prospective assessment of cytosolic DNA burden, STING pathway activation, and downstream IFN-I output in tumor and peripheral blood is needed to define responsive populations and move from empirical combinations toward biomarker-driven selection.

### Viral vectors and oncolytic/immune-stimulating platforms.

Oncolytic viruses (OVs) and viral vectors represent an alternative strategy for engaging innate immune sensing networks through viral replication, immunogenic cell death, and release of pathogen- and damage-associated molecular patterns, rather than direct pharmacologic STING agonism (135, 136). This distinction has therapeutic implications. OV susceptibility is often inversely correlated with tumor STING competence, as intact IFN-I responses can restrict viral replication. Conversely, tumors with impaired STING/IFN signaling may permit more efficient viral propagation while remaining capable of supporting immune priming through stromal and DC-mediated sensing (137). In contrast to direct agonists, which require preserved pathway signaling for activity, OV platforms may exploit tumor-intrinsic pathway deficiencies while activating innate networks in the TME.

As highlighted in a comprehensive review by Davola and Mossman (138), induction of immunogenic cell death and activation of host antitumor immunity frequently represent the dominant drivers of therapeutic benefit, even in settings of limited productive replication. Consistent with this paradigm, studies using modified vaccinia virus Ankara (MVA) have shown that viral replication is dispensable for systemic antitumor immunity. Intratumoral delivery of heat-inactivated MVA induces robust IFN-I production via the cGAS/STING pathway and generates adaptive antitumor responses that depend on BATF3-dependent CD103^+^CD8α^+^ DCs (139). Direct comparisons further demonstrated that heat-inactivated vaccinia induces stronger IFN-I production and superior systemic antitumor immunity compared with replication-competent virus (140). These findings support a model in which innate sensing within tumor-resident immune cells, rather than tumor-intrinsic viral cytolysis, governs therapeutic efficacy.

Building on this mechanistic framework, recombinant MVA platforms have been engineered to enhance innate sensing and adaptive priming through deletion of viral immune evasion genes — including the cGAS inhibitor E5R — and expression of immunostimulatory ligands (141). Preclinically, MQ710 (MVAΔE5R-Flt3L-OX40L) generates potent antitumor immunity that depends on CD8^+^ T cells, tumor cGAS/STING-mediated DNA sensing, and IFN-I signaling, while simultaneously depleting immunosuppressive OX40^hi^ Tregs within the injected tumor through combined OX40L-OX40 and IFNAR-dependent mechanisms (141). This dual action illustrates how a viral platform can be engineered to engage the immunogenic cGAS-STING-IFN-I axis while redirecting the same IFN-I output toward relief of intratumoral immunosuppression. A first-in-human phase I study is currently evaluating intratumoral administration of humanized recombinant MVA MQ710 in patients with advanced solid tumors (NCT05859074).

Collectively, these findings support a revised model of viral immunotherapy in which selective activation of innate sensing pathways within the TME, particularly DC-dependent cross-priming, serves as the central mechanistic axis of therapeutic response. In this framework, viral replication may augment antigen release and inflammatory signaling but is not strictly required, provided that innate immune activation and adaptive priming remain intact.

Talimogene laherparepvec (T-VEC), an HSV-1–based OV-expressing GM-CSF, is FDA approved for unresectable melanoma (142). T-VEC induces immunogenic cell death characterized by HMGB1 release, ATP secretion, and calreticulin exposure, promoting local and systemic antitumor immunity (137). Combination strategies have yielded variable results. A phase II trial combining T-VEC with the checkpoint inhibitor ipilimumab improved ORR compared with ipilimumab alone, whereas in the double-blind, placebo-controlled phase III MASTERKEY-265 trial of T-VEC plus pembrolizumab did not improve progression-free or overall survival relative to pembrolizumab monotherapy (143, 144).

Beyond melanoma, the Pexa-Vec (JX-594) GM-CSF–armed OV produced a dose-dependent overall survival benefit in a randomized phase II trial in advanced hepatocellular carcinoma (145). However, the subsequent phase III PHOCUS trial of sequential Pexa-Vec followed by sorafenib versus sorafenib alone in advanced hepatocellular carcinoma was terminated early at interim futility analysis, without improvement in overall survival (146). The negative phase III outcome likely reflects multiple factors, including pharmacological antagonism between sorafenib-induced cell-cycle arrest and the cell-cycle–dependent replication of TK1-deleted vaccinia and the limited capacity of unmodified vaccinia to engage cGAS/STING signaling owing to viral immune-evasion gene products (63, 147, 148). This mechanistic constraint provides direct rationale for next-generation recombinant MVA platforms such as MQ710, in which deletion of viral immune-evasion genes restores cGAS-STING-IFN-I signaling (141).

These findings suggest that virus-mediated innate activation alone may be insufficient to overcome established resistance in unselected populations. The contrasting outcomes of T-VEC plus ipilimumab versus T-VEC plus pembrolizumab may also reflect the distinct mechanisms of these checkpoint inhibitors (149). Anti–CTLA-4 antibodies act largely at the priming phase by relieving CTLA-4–mediated suppression and depleting intratumoral Tregs through Fc-dependent mechanisms (149, 150) and may therefore synergize particularly well with the innate immune-driven priming generated by intratumoral T-VEC. In contrast, anti–PD-1 acts predominantly at the effector phase by reinvigorating exhausted CD8^+^ T cells already in the tumor (149), an axis less dependent on additional priming.

Oncolytic herpes simplex virus platforms have advanced in glioblastoma. G47Δ (teserpaturev/Delytact) was approved in Japan, under conditional and time-limited authorization, for malignant glioma. An initial phase I/II study reported a median overall survival of 7.3 months in heavily pretreated recurrent glioblastoma, and the subsequent pivotal single-arm phase II trial in residual or recurrent glioblastoma met its primary endpoint with a 1-year survival rate of 84.2% and a median overall survival of 20.2 months (151). Radiographic “immunoprogression,” reflecting immune infiltration rather than tumor growth, has been described following treatment. Posttreatment biopsies demonstrated tumor cell destruction and lymphocyte infiltration consistent with engagement of innate sensing pathways, although the specific contribution of cGAS/STING signaling was not directly quantified in patients (151, 152).

Adenoviral platforms have similarly progressed clinically. DNX-2401 combined with pembrolizumab in recurrent glioblastoma (CAPTIVE/KEYNOTE-192) demonstrated feasibility and durable disease control in a subset of patients (153). In urothelial carcinoma, CG0070 (cretostimogene grenadenorepvec, a GM-CSF–expressing oncolytic adenovirus) has shown activity in BCG-unresponsive non-muscle-invasive bladder cancer (NMIBC), with a phase II study demonstrating a 47% complete response rate at 6 months in patients with carcinoma in situ (CIS) (154). In the CORE-001 trial combining CG0070 with pembrolizumab, the 12-month CR rate was 57.1% and the 24-month CR rate was 51.4%, with no progression to muscle-invasive disease (155). Nadofaragene firadenovec (rAd-IFNα/Syn3), a nononcolytic adenoviral vector expressing IFNα2b, achieved a 53.4% complete response rate at 3 months in BCG-unresponsive CIS and is FDA-approved (156).

A potential limitation of STING-centered therapeutic strategies is that tumor-intrinsic defects in cGAS/STING signaling, or ENPP1 upregulation, can impair cytosolic DNA sensing and IFN-I production, limiting DC activation and T cell priming (157, 158). Viral platforms are distinctively positioned with respect to this limitation. Because OVs and viral vectors engage multiple innate sensors in parallel, they can sustain IFN-I induction and immune priming even when tumor-intrinsic cGAS/STING signaling is compromised (158). This constitutes a key conceptual contrast with direct STING-centered approaches: whereas STING agonists require an intact, signaling-competent pathway to achieve durable priming, viral platforms can convert STING pathway deficiency from a liability into a therapeutic opening by recruiting parallel sensing networks within the TME.

Preclinical studies have explored combining OVs with STING agonists. In glioblastoma models, oncolytic HSV combined with membrane-impermeable 2’3’-cGAMP enhanced CD8^+^ T cell activation and DC priming without compromising viral replication, consistent with tumor-intrinsic STING pathway deficiencies (159). These data support the concept that viral platforms and pharmacologic STING activation may be complementary rather than antagonistic under defined biological conditions.

Collectively, OV and viral vector programs demonstrate that clinically meaningful immune activation can emerge from coordinated innate sensing networks in which STING operates as one component of a broader inflammatory cascade. As with direct agonists and DNA damage-based strategies, therapeutic durability appears to depend on baseline tumor immune context, functional pathway competence, and rational combination design. Integration of viral platforms with biomarker-driven patient selection and immune checkpoint blockade may help better define the therapeutic window for STING-centered immunomodulation.

## Conclusion

The cGAS/STING pathway has emerged as a central molecular interface linking genome instability to immune regulation in cancer, yet its functions cannot be reduced to a binary model of tumor suppression versus tumor promotion. Rather, accumulating evidence across tumor and immune compartments supports a framework in which cGAS/STING signaling operates within distinct biological regimes defined by activation duration, cellular localization, and downstream transcriptional bias. Acute, spatially constrained activation, often triggered by transient DNA damage or immunogenic cell death, favors IFN-I–dominated programs that license DC cross-priming, sustain cytotoxic lymphocyte function, and promote antitumor immunity. In contrast, chronic activation driven by persistent chromosomal instability and mislocalized self-DNA reprograms pathway output toward stress-adaptive, inflammatory, and immunosuppressive states that facilitate tumor progression, metastatic dissemination, and immune dysfunction.

This context dependence is reinforced by multilayered spatial and biochemical regulation of pathway components. Nuclear tethering, posttranslational modification, and phase separation constrain cGAS activity under homeostatic conditions, while regulated STING trafficking and degradation impose temporal limits on downstream signaling. When these safeguards erode under sustained genomic stress, cGAS/STING signaling acquires noncanonical functions, including IFN-independent effects on DNA repair, ER stress–associated inflammatory rewiring, and deleterious effects on lymphocyte viability and differentiation. Thus, a pathway that initiates immune surveillance can, under chronic activation, be co-opted to fuel tumor evolution and immune evasion.

These mechanistic insights carry important implications for therapeutic development. Early clinical experience with direct STING agonists has underscored the liabilities of indiscriminate pathway activation, including limited durability and systemic toxicity. Emerging strategies increasingly emphasize modulation rather than maximal activation, amplifying endogenous DNA sensing, restricting signaling to defined cellular compartments, or enhancing paracrine cGAMP transfer while preserving spatial control. Viral and DNA damage–based platforms further illustrate that productive engagement of cGAS-STING often arises from coordinated innate sensing networks rather than isolated pharmacologic agonism.

Looking forward, clinical translation will require shifting from pathway activation as an endpoint to pathway regulation as a guiding principle. Identifying biomarkers that distinguish beneficial from maladaptive signaling states, defining cell type–specific tolerances to STING activation, and integrating pathway tuning with immune checkpoint blockade and genotoxic therapies represent key priorities. The central challenge is no longer whether cGAS-STING should be activated or inhibited in cancer, but when, where, and to what extent it should be engaged to harness its immunostimulatory capacity while avoiding its tumor-promoting consequences.

## Conflict of interest

Memorial Sloan Kettering Cancer Center has filed a patent application for the use of MQ710 (MVAΔE5R-Flt3L-OX40L) as monotherapy or in combination with immune checkpoint blockade for the treatment of solid tumors, on which LD and YW are listed as inventors.

## Funding support

This work is the result of NIH funding, in whole or in part, and is subject to the NIH Public Access Policy. Through acceptance of this federal funding, the NIH has been given a right to make the work publicly available in PubMed Central.

- Center for Experimental Therapeutics at Memorial Sloan Kettering Cancer Center (to LD).
- The Rockefeller University Center for Clinical and Translational Science Pilot Awards (to JAL and LD).
- JAL is a clinical scholar at Rockefeller University.
- NIH/National Cancer Institute Cancer Center Support Grant P30 CA008748.
- Melanoma Research Alliance Pilot Award (to LD).

## Figures and Tables

**Table 1:**
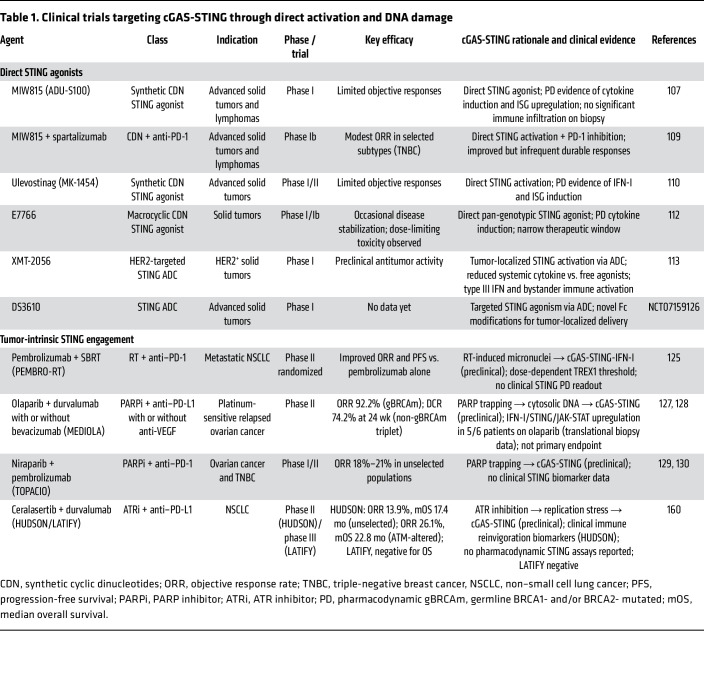
Clinical trials targeting cGAS-STING through direct activation and DNA damage

**Table 2:**
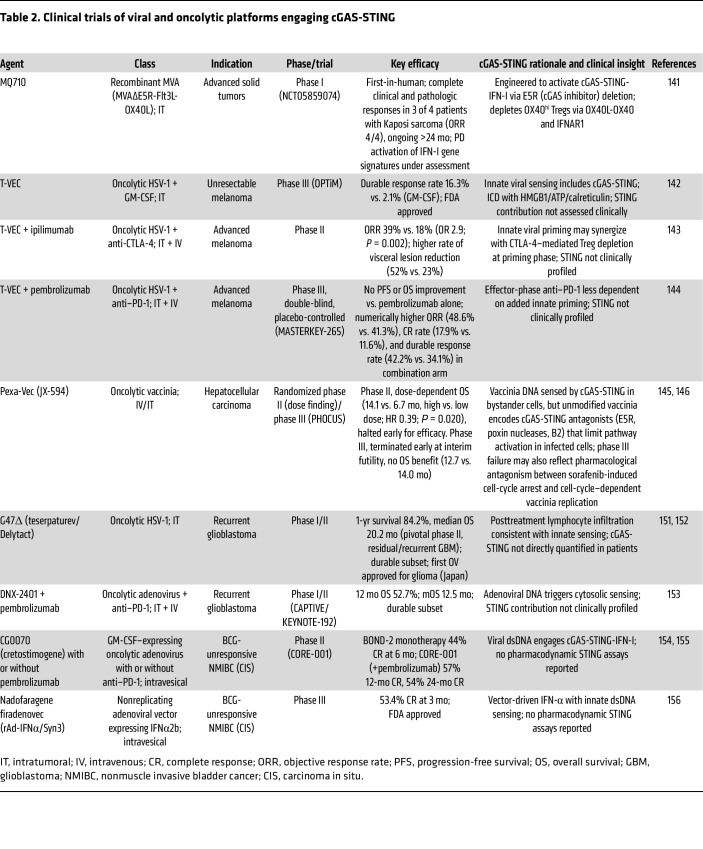
Clinical trials of viral and oncolytic platforms engaging cGAS-STING

## Version 1 08/03/2026

Electronic publication
